# Adapting segment anything model for hematoma segmentation in traumatic brain injury

**DOI:** 10.1007/s44352-025-00011-4

**Published:** 2025-05-26

**Authors:** Lingrui Cai, Craig Williamson, Andrew Nguyen, Emily Wittrup, Kayvan Najarian

**Affiliations:** 1 Department of Computational Medicine and Bioinformatics, University of Michigan, 2800 Plymouth Road, Ann Arbor, 48109 MI USA; 2 Department of Neurosurgery and Neurology, University of Michigan, 1500 E. Medical Center Drive, Ann Arbor, 48109 MI USA; 3 Michigan Institute for Data Science, University of Michigan, 500 Church Street, Ann Arbor, 48109 MI USA; 4 Max Harry Weil Institute for Critical Care Research and Innovation, University of Michigan, 2800 Plymouth Road, Ann Arbor, 48109 MI USA

**Keywords:** Traumatic brain injury, Segment anything model, Neurology, Hematoma segmentation, Artificial intelligence, Parameter efficient transfer learning, Adapter

## Abstract

Hematoma segmentation in traumatic brain injury (TBI) is critical for accurate diagnosis and effective treatment planning. In this study, we evaluate various automated segmentation models, including stat-of-the-art architecture as benchmarks, and compare their performance with our proposed SAM-Adapter method for segmenting hematomas in brain CT scans. By incorporating the adapter into the vanilla SAM model, we address the challenges in medical imaging, which has very limited annotated datasets, enhancing model performance efficiency. We also find that domain-specific pre-processing, such as contrast adjustment, reduces the need for extensive pretraining, making the model more streamlined. And the model performance benefited with optimization and hyperparameter tuning. Our results demonstrate that the SAM-Adapter model achieved strong performance and reliability in identifying hematomas with Dice (72.34%), IoU (59.78%), 95% HD (5.57), sensitivity (75.39%) and specificity (99.73%). Inter-observer variability was assessed, revealing that the model’s performance Dice (67.20%) was closely aligned with human expert agreement Dice (63.79%), suggesting its potential clinical utility. The external validation on the HemSeg-200 dataset, which contains 222 scans, demonstrates the robustness of our approach across diverse cases. These advancements in automatic segmentation hold promise for improving the accuracy and efficiency of TBI diagnosis, supporting clinical decision-making, and enhancing patient outcomes.

**Supplementary Information:**

The online version contains supplementary material available at 10.1007/s44352-025-00011-4.

## Introduction

Traumatic Brain Injury (TBI) is the most prevalent neurological disorder, representing a significant public health challenge with an estimated 50–60 million new cases each year [1]. TBI results from an external force causing damage to the brain, leading to various degrees of physical, cognitive and emotional impairments. One of the critical complications of TBI is the formation of hematomas, which involves the accumulation of blood clots within the brain tissues [2, 3]. The type and volume of hematomas are critical in predicting TBI outcomes, as delayed management can lead to increased intracranial pressure, neurological deficits, unconsciousness, and death[4]. Effective assessment and intervention during the critical "golden hours" following the injury are crucial yet challenging[5].

Computed tomography (CT) scans are the gold standard for TBI assessment, especially hematoma detection and evaluation in emergency settings[6]. Accurate and timely hematoma estimation is essential for effective clinical management of TBI patients, as it enables precise measurement of lesion volume and location, potentially aiding in prioritizing cases and applying treatment[7, 8]. However, manual analysis of CT images by radiologists can be time-consuming, labor-intensive, and subject to inter-observer variability. There is a growing need for automated systems capable of efficiently analyzing CT data to estimate brain injury extent and provide prognostic information. Such systems could standardize TBI management, reduce human error, expedite decision-making, and tailor treatments based on individual patient profiles[9]. This need is particularly acute in smaller hospitals lacking specialized expertise, where TBI incidence and mortality are higher.

Recent advancements in medical image analysis, particularly with the advent of deep learning techniques, have shown promise in automating hematoma detection and segmentation[10]. Convolutional neural networks (CNNs) have been extensively used due to their powerful feature extraction capabilities. Architectures such as U-Net and its variants have become popular for medical image segmentation tasks. U-Net’s encoder-decoder structure allows for precise localization and boundary delineation of hematomas[11, 12]. Enhancements like attention mechanisms and multi-scale feature integration have further improved segmentation accuracy by enabling the network to focus on relevant regions and capture contextual information at different scales[13, 14]. Combining CNNs with other computer vision techniques, such as hand-craft features, has been explored to enhance segmentation performance[15]. These hybrid models leverage the strengths of different approaches to improve robustness and accuracy. Moving from 2D to 3D CNNs has allowed for better utilization of volumetric information inherent in CT scans. 3D networks capture spatial relationships more effectively and lead to improved segmentation of complex structures like hematomas[16–18].

The Segment Anything Model (SAM) has revolutionized computer vision, especially in image segmentation, by demonstrating robust zero-shot generalization and handling a variety of object segmentation tasks. SAM outperforms conventional models that struggle with intricate boundaries, low contrast, and complex scenes by using advanced vision transformer techniques and simple prompts like points, boxes, and coarse masks[19]. This innovation has attracted significant attention in medical image segmentation, where SAM is adapted for various applications. Foundational models fine-tune SAM parameters with extensive medical image datasets, integrating multiple segmentation tasks into a unified framework despite high computational demands [20, 21]. Recently, parameter-efficient transfer learning through adapter tuning[22] has emerged, inserting adapter modules into transformer blocks and updating only the adapters during training while keeping the original parameters partially frozen, proving to be effective[23].

This study proposes to effectively adapt the SAM framework and parameter-efficient fine-tuning for hematoma segmentation in TBI. Our experimental design encompasses the optimization of loss functions, specifically balancing binary cross-entropy and boundary loss, and evaluating the dimension of the adapter block. Additionally, we incorporated annotations from multiple reviewers to prove model generalization and robustness. We also explored the effects of pretraining the model with public datasets. These approaches achieved superior evaluation scores that enhanced the automatic measurement of hematoma volume and accelerated the TBI diagnosis process. These advancements aim to address current clinical challenges and improve outcomes in TBI management by providing reliable and efficient tools for hematoma segmentation.

## Methods

### CT representation and prepossessing

This study employs non-contrast brain CT scans as its input. The CT images are acquired in axial view, capturing the anatomical structure of the brain tissue with high spatial resolution. These images are all represented in a standardized format, as Digital Imaging and Communication in Medicine (DICOM) files, which preserve important metadata regarding patients’ information, acquisition parameters, and resolution. We assume that the input images $x \in X$, where $X$ represents the input space. Each CT scan $x \in \mathbb R^{H\times W \times C}$ represents a single CT scan voxel, with *H* and *W* denoting the height and width of one slice of the scan, and *C* denoting the total number of slices stacked in the scan.

#### Conversion to Hounsfield units(HU)

The first preprocessing step is to convert the gray values stored in the DICOM format file to Hounsfield Units[24] using a linear transformation, which can be defined as:

1 $$\begin{aligned} x_{HU} = x \times slope + intercept.\end{aligned}$$

where $x_{HU}$ is the transformed image in Hounsfield Units, and the parameters slope and intercept are the rescale slope and rescale intercept retrieved from the DICOM header.

#### Contrast adjustment

Contrast adjustment remaps image intensity values to the full presentation range of the interesting tissue type. An image with good contrast is expected to have sharp differences between black and white. The contrast of the CT image is adjusted by selecting a range of HU values[$lower, upper$]. This adjustment aims to enhance the visibility of the specific anatomical structure or pathological conditions within the brain. The contrast adjustment function is defined as follow:

$$f(x) = {\left\{ \begin{array}{ll} 0 & \text {for } x_{HU} \le lower \\ 255 \times \dfrac{x_{HU} - lower}{upper - lower} & \text {for } lower< x_{HU} < upper \\ 255 & \text {for } x_{HU} \ge upper \end{array}\right. }$$

**Fig. 1:**
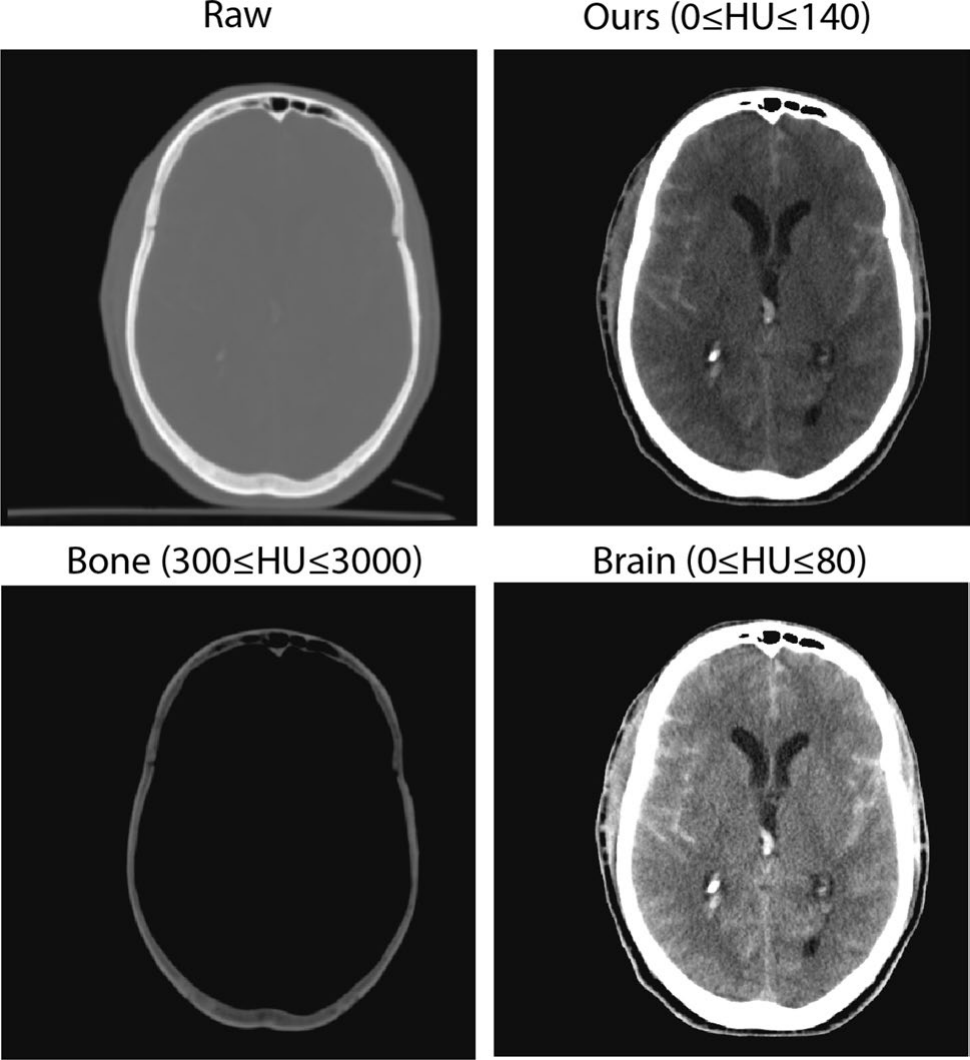
Contrast adjustment of CT scans with different window width settings

The commonly used range of HU values for brain CT studies is [0, 80]. However, in this study, the hematoma pathological tissue we are focusing on is brighter than normal brain tissue. We adopt range [0, 140] in this study as a baseline contrast adjustment. (Fig. 1)

#### Image transformation

After contrast adjustment, the orientation of the 3D brain image is calculated, and affine transformation is applied based on the calculated angle to ensure uniform orientation across all the cases. To standardize the slices into a fixed size, 0 pads to the margins for those images with slices height/width smaller than 512, and cropping applies to the slices height/width larger than 512.

### Segment anything architecture

**Fig. 2:**
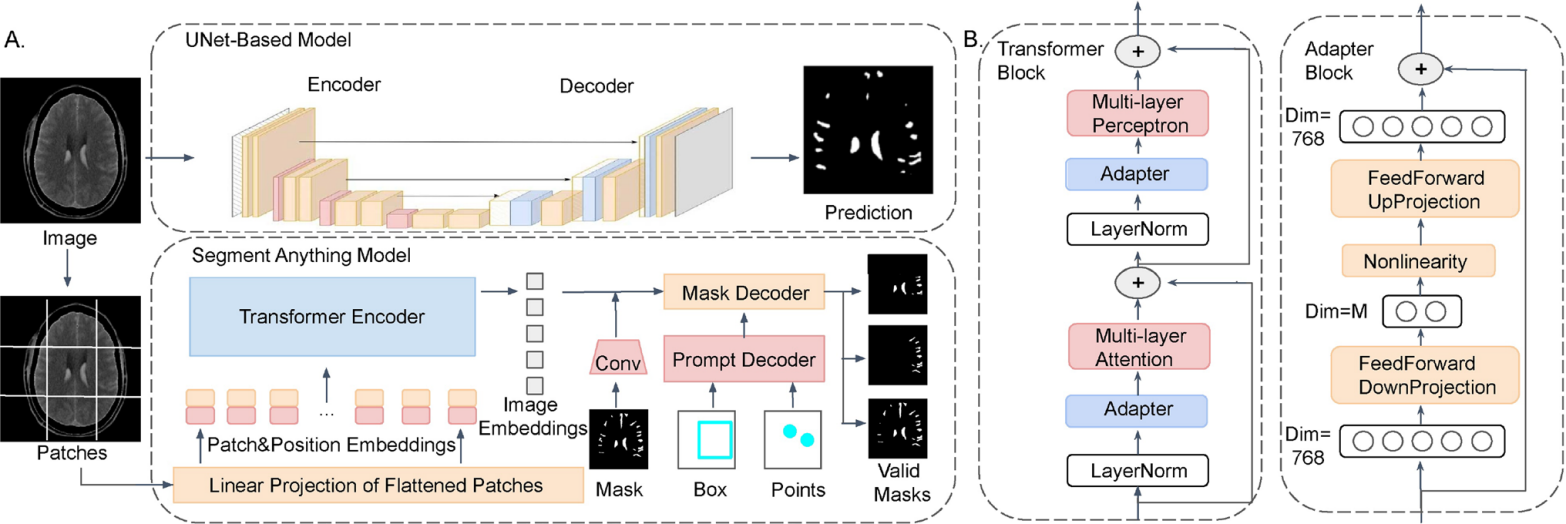
Model architecture overview. ** A** The SAM architecture with selected prompt.** B** The transformer block in image encoder and Adapter block inside the transformer block

The SAM architecture[19] is delineated by three principal components: the image encoder, the prompt encoder, and the mask decoder. The image encoder utilizes a standard Vision Transformer (ViT)[25] pre-trained using Masked Auto-Encoders (MAE)[26]. Specifically, the ViT-B/16 base model is employed in this study, featuring $16\times 16$ fixed-size patches. These patches are linearly embedded and augmented with position embedding, then passed through a standard transformer encoder with four uniformly distributed global attention blocks, as illustrated in Fig. 2.

The prompt encoder can operate in either a sparse (points, boxes) or dense (masks) mode. This study focuses on the sparse encoder, which encodes points and boxes through positional encoding integrated with learned embedding for each prompt type. The mask decoder comprises a Transformer decoder block, adapted to include a dynamic mask prediction head. This decoder utilizes two-way cross-attention to capture the interactions between the prompt and image embedding. Subsequently, SAM enhances the resolution of the image embedding, and a Multi-layer Perceptron (MLP) translates the output token into a dynamic linear classifier, ultimately predicting the target mask for the given image.

### Parameter-efficient transfer learning

Traditional fine-tuning of large pre-trained models is a well-established transfer mechanism in biomedical image segmentation[27, 28]. However, it becomes parameter inefficient and may suffer from noticeable performance drop when fine-tuned on large foundation model with limited image dataset availability. As more efficient alternative, freezing or fine-tuning specific layers, has emerged and became compact and extensible solution[29, 30].

Parameter-efficient transfer learning(PETL) is a cutting-edge technique in machine learning, designed to adapt or fine-tune pre-trained models for a target task while adding minimal additional parameters[22]. This approach capitalizes on the knowledge obtained from a source task to enhance performance on a related but distinct target task. Within this paradigm, adapter tuning emerges as a pivotal strategy, involving modifications to specific components of the pre-trained model to align better with the target task, all while minimizing the introduction of new parameters. This approach aims to achieve a balance between efficient model transfer and task-specific optimization, thereby boosting the performance and conserving computational resources and have been widely applied in the pre-train vision models, especially in medical images[31, 32]. Related work also immerged fast recently with adaption to SAM model for underperformed scences [33], general medical images [23] and tailored for intracranial hemorrhage segmentation [34]

In this study, we implement serial adapter [22] by strategically inserting adapter blocks immediately after two primary sub-layers, the multi-layer attention and multi-layer perceptron, in each transformer layer. The details of this approach are demonstrated in Fig. 2B. To limit the number of parameters, we use a widely employed bottleneck architecture that maintains the pre-trained backbone unchanged and introduces only a minimal number of trainable parameters. Specifically, the adapter projects the original d-dimensional features into a smaller dimension m with a down-projection matrix $W_{\text {down}} \in \mathbb R^{d\times m }$, followed by a non-linear activation function ReLU, and then projects them back to d-dimension space with up-projection matrix $W_{\text {up}} \in \mathbb R^{m\times d }$.

2 $$\begin{aligned} \text {Adapter}(x) = W_{\text {up}} (ReLU (W_{\text {down}} x) )+ x\end{aligned}$$

### Optimizations and hyperparameter tuning

To enhance the performance of our hematoma segmentation models, several optimizations and hyperparameter tuning strategies were employed in this study. A key focus was on the loss function, where we experimented with different loss functions and combinations. By fine-tuning the combination ratio, we aimed to balance pixel-wise classification accuracy with precise boundary delineation, ultimately improving segmentation quality. Additionally, the dimension m of the adapter blocks within the PETL Adapter unit was carefully selected. Various values of m were tested to determine the optimal size that maximizes model efficiency and performance without significantly increasing computational complexity. Furthermore, we leveraged public hemorrhage datasets as pretraining datasets to initialize our models, allowing them to learn robust and generalizable features before fine-tuning on the specific hematoma segmentation task. This kind of pretraining step was involved in state-of-the-art models and aimed to improve convergence rates and overall accuracy. Lastly, contrast adjustment was applied to the input CT slices, standardizing the window level to [0–140] HU. This preprocessing step enhanced the visibility of hematomas, making it easier for the models to learn relevant features during training.

### Benchmark methods

#### U-Net

The U-Net architecture is a fully convolutional neural network designed for biomedical image segmentation[35]. It follows an encoder-decoder structure, where the encoder progressively reduces the spatial dimensions of the input image through a series of convolutional and pooling layers, capturing context and essential features. The decoder then reconstructs the spatial dimensions through upsampling and convolutional layers, utilizing skip connections from the encoder to preserve fine-grained details and improve segmentation accuracy.

#### Multi-view CNN

Multi-view CNN is an architecture proposed to improve brain acute hematoma segmentation on CT scans from patients with TBI based on U-Net [12]. It extracted features from different scales and fused them to improve the segmentation performance. It’s a novel method that could decouple features for generating segmentation masks and features for hematoma identification. Our results show that the network can lead to both a finer hematoma segmentation and better hematoma identification accuracy.

#### nnU-Net

The nnU-Net proposes an automated framework, instead of novel neural network, for biomedical image segmentation[36]. It dynamically configures its architecture and training protocols based on the properties of the input dataset, ensuring optimal performance without manual intervention. nnU-Net extends the traditional U-Net design by incorporating extensive pre-processing, data augmentation, and post-processing steps, while also adapting the network architecture, including depth, kernel size, and feature map dimensions, to suit the specific characteristics of the data. In this study, we utilize the 3D full-resolution configuration of nnU-Net as a benchmark for 3D segmentation performance.

**Table 1:** Dataset statistics for the experimental dataset and pre-training dataset

	Dataset	Type	Scan(Pos)	Scan(Neg)	Slice(Pos)	Slice(Neg)	25th*	50th*	75th*
Experimental	PROTECH III[37]	Hematoma	71	0	1345	1718	687	1835	3621
Experimental	UMICH	Hematoma	28	25	476	1334	1854	3542	5124
Pre-training	BHSD[38]	Hemorrhage	192	0	2368	4036	347	1081	2971
Pre-training	INSTANCE[39]	Hemorrhage	100	0	893	2088	537	1498	3866
Pre-training	PhysioNet[40]	Hemorrhage	75	0	332	2482	520	1103	2231
Validation	HemSeg-200[41]	Hemorrhage	222	0	1677	5658	431	1148	2492

25th*, 50th* and 75th* present for the quartile analysis for the number of positive pixels in positive slices

## Experiments

### Datasets

#### Experimental dataset

The experimental dataset used in this study comprises a collection of brain CT images specifically curated for traumatic brain injury(TBI). This dataset consists of 119 CT scans obtained from Progesterone for traumatic brain injury, Experimental Clinical Treatment (PROTECT III) clinical trial [37], and the University of Michigan. It covers a diverse range of cases, CT scanners, and protocols to ensure robust model evaluation.

The PROTECT III clinical trial was conducted at 49 trauma centers across the United States. It included adult patients with moderate to severe TBI, with a Glasgow Coma Scale(GCS) score ranging from 4 to 12. The University of Michigan dataset was collected from the General Neurology Clinic and Emergency Department of the University of Michigan.

In total, there are 94 CT scans with hematoma findings, which serve as positive samples, and 25 CT scans without hematoma, which constitute the negative pool (Table 1). Each image has been annotated by an expert neurologist with subspecialization in neurocritical care (Reviewer A), providing ground truth masks for accurate assessment of segmentation performance. Additionally, a subset of the image has been independently annotated by another expert neurologist and neurocritical care specialist(Reviewer B), who did not have access to the annotations made by neurologist A and did not discuss the imaging with A. The subset of double-reviewed annotations serves as additional validation in this study.

#### Pre-training dataset

Given the absence of publicly available traumatic hematoma segmentation datasets, we acquired three public pixel-level intracranial hemorrhage segmentation datasets for model pretraining purposes. These include the Brain Hemorrhage Segmentation Dataset (BHSD) with 192 CT scans [38], the 2022 Intracranial Hemorrhage Segmentation Challenge (INSTANCE 2022) with 100 CT scans[39, 42], and the PhysioNet Intracranial Hemorrhage Detection and Segmentation challenge with 82 CT scans [40] (Table 1). These images cover various types of hemorrhage in different anatomical regions.

#### External validation dataset

To evaluate the generalizability of our model, we conducted external validation using the HemSeg-200 dataset[41], which was sourced from RSNA(RSNA Intracranial Hemorrhage Detection)[43] and was meticulously annotated at the voxel level for precise hemorrhage. This dataset consists of 222 scans from different patients, covering a diverse range of Intraparenchymal hemorrhage (IPH) and intraventricular hemorrhage (IVH).

### Evaluation metrics

Given the focus of this work on hematoma segmentation, we have selected the Dice Similarity Coefficient (Dice), Intersection over Union (IoU), the 95th percentile of the Hausdorff Distance (95%HD), Accuracy, Sensitivity, and Specificity as metrics to evaluate the segmentation effectiveness across various dimensions [44] in the slice level. For scan-level evaluation, we introduce Volumetric Similarity (VS)[45] to measure the volume difference between predictions and ground truth. In addition, Dice, IoU and 95%HD are computed in 3D as volume-based metrics. Dice and IoU quantify the overlap between the predicted (P) regions and ground truth (GT) masks at the pixel level, and 95% HD measures how far two subsets of a metric space are from each other. They are computed by the following equations:

3 $$\begin{aligned} Dice= & \frac{2|P \cap GT|}{|P| + |GT|}\end{aligned}$$

4 $$\begin{aligned} IoU= & \frac{|P \cap GT|}{|P \cup GT|}\end{aligned}$$

5 $$\begin{aligned} HD= & \max \{dist(P,GT),dist(GT, P)\}\end{aligned}$$

6 $$\begin{aligned} VS= & 1 - \frac{||P| - |GT||}{|P| + |GT|} \end{aligned}$$

### Implementation details

For model training and validation, we hold 20% of the images as the test set, while the remaining images were divided into the training and validation sets using a 5-fold cross-validation schema. To balance the data distribution, positive images and negative images were stratified split to ensure similar ratio of hematoma cases in each set. Each image underwent normalization using the min-max method. Data augmentation techniques, including random rotation, random zoom, random horizontal flip, random shift, and color jitter, were applied with a probability of 0.5 to the training dataset before being fed into the network.

For the experimental dataset, slice-level evaluation metrics are reported as the mean of the fold-wise means ± the standard deviation of the fold-wise means and the scan-level evaluation metrics are reported as the mean of the fold-wise means ± the mean of the fold-wise standard deviation. For the external evaluation dataset, the same preprocessing and evaluation pipeline were applied to ensure consistency with the original report. Performance metrics were reported at the scan level only with bootstrapping, along with 95% confidence intervals.

The SAM model was initialized with pre-trained weights from the Segment Anything 1 Billion (SA-1B) dataset (model weights from: https://huggingface.co). All models underwent fine-tuning using our hematoma experimental dataset over 50 epochs. Optimization was performed using the AdamW optimizer, with a learning rate of 5e-5, a decay rate of 0.1, and a batch size of 2. Binary Cross Entropy (BCE) was used as the default loss function. Additionally, some experiments were conducted using Dice Loss, Focal Tversky Loss, and a combination of Boundary Loss (BL) with BCE/Dice. The experiments were conducted on NVIDIA Tesla V100 and NVIDIA A40 GPUs.

### Statistical analysis of model performance

To assess whether the different optimization strategies lead to significant performance improvements, we employed Friedman’s test[46], a non-parametric statistical test designed for comparing multiple related samples. This test is particularly suitable for evaluating model performance across multiple datasets or experimental conditions, as it does not assume normality in the data distribution.

Friedman’s test ranks the performance of each model within each dataset and then determines whether the observed differences in rankings are statistically significant. If the test results indicate a significant difference (i.e., p-value< 0.05), we conduct a post-hoc Nemenyi test to perform pairwise comparisons and identify which model or optimizations setting yields significantly different performance levels. If no significant difference is detected, we conclude that the optimizations do not provide statistically distinguishable improvements.

## Results

### Hematoma segmentation performance comparison

The segmentation performance of various models for hematoma segmentation in TBI was evaluated in Table 2 for the slice-level performance and Table S1 for the scan-level performance. The UNet model achieved a Dice coefficient of 56.40% and an IoU of 44.00%. The MultiView approach exhibited performance similar to UNet, with a Dice of 56.60% and an IoU of 44.40%. Those two models demonstrated high specificities, which indicated low false positive rate. However, the sensitivity was relatively low, suggesting limitations in detecting all positive cases and a higher false negative possibility. The nnUNet significantly outperformed both UNet and MultiView, achieving a Dice of 65.02% and an IoU of 62.76%. It had a high sensitivity of 88.03%, reflecting its effectiveness in identifying hematomas. The model also exhibited a lower 95%HD of 5.89, indicating improved boundary accuracy. The SAM model showed the lowest performance among the evaluated models. The proposed SAM-Adapter model achieved the highest performance across most metrics. It attained slice-level Dice coefficient of 72.34%, an IoU of 59.78% and the lowest 95%HD of 5.73. For the scan level evaluation, it has consistent superior performance with 75.51% Dice and 92.05% VS, which indicating superior boundary delineation.

To assess the significance of differences in model performance, we conducted Friedman’s test on scan-level evaluations across different model architectures. The results yielded a p-value below 0.05 for Dice, IoU, 95%HD, and VS, indicating statistically significant differences among the models. Following this, a Nemenyi post-hoc analysis was performed with visualized results in Table S3, with detailed results presented in Figure S1. The analysis revealed no significant difference (NS) between the UNet and MultiView models. However, nnUNet demonstrated a clear improvement in performance, and the SAM-Adapter method exhibited an additional performance gain.

**Fig. 3:**
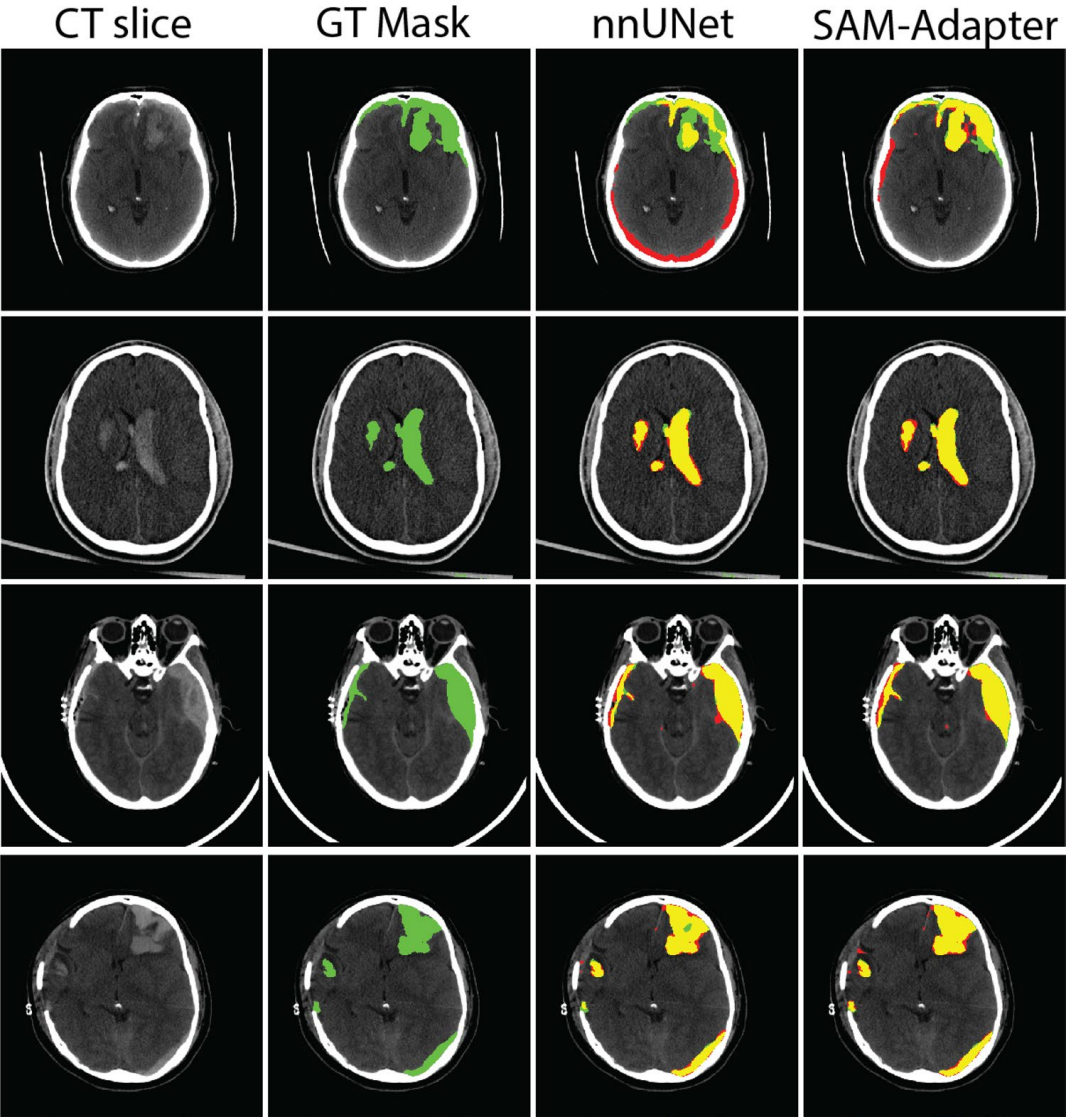
Performance comparison between difference models. Each row represents a selected slice from the test dataset. The first column shows the contrast-adjusted CT slices with a window level of [0–140] HU. The second column displays the Ground Truth (GT) masks manually annotated by Reviewer A (in green). The third and fourth columns present the segmentation results obtained using the nnUNet and SAM-Adapter methods, respectively. The intersecting regions, where the model predictions agree with the GT, are shown in yellow. Non-overlapping pixels retain their original colors (green for GT and red for model predictions)

Overall, our proposed SAM-Adapter model demonstrated the best performance, significantly outperforming other models in terms of Dice coefficient, IoU, sensitivity, 95%HD and VS, while maintaining high accuracy and specificity. This suggests that the SAM-Adapter model provides a more reliable and precise segmentation of hematomas in CT images of TBI patients. The selected inference results are visualized in Fig. 3 to provide an intuitive comparison of segmentation performance.

### SAM-adapter model optimization and hyperparameter tuning

In this section, we conducted extensive experiments to optimize the performance of our segmentation models. We focused on the loss functions, the dimension M of adapter blocks, and the impact of pretraining datasets. The detailed results are summarized in Table 3

**Table 2:** Segmentation performance comparison across different models. All models were trained using a 5-fold cross-validation schema, and slice-level evaluation metrics are reported as the mean of the fold-wise means ± the standard deviation of the fold-wise means

Model	Dice(%)	IoU(%)	95%HD	Accuracy	Sensitivity	Specificity
UNet [35]	56.40 ± 3.85	44.00 ± 3.32	6.24 ± 0.13	99.00 ± 0.00	56.00 ± 5.79	100.00 ± 0.00
MultiView [12]	56.60 ± 3.78	44.40 ± 3.46	6.24 ± 0.12	99.00 ± 0.00	55.20 ± 5.26	100.00 ± 0.00
nnUNet [36]	65.02 ± 0.45	62.76 ± 2.85	5.89 ± 0.06	99.69 ± 0.02	88.03 ± 0.31	99.82 ± 0.02
SAM [19]	44.14 ± 5.36	31.46 ± 4.65	6.95 ± 0.11	98.90 ± 0.06	45.93 ± 11.14	99.61 ± 0.16
SAM-adapter	${\textbf {72.34}}\pm {\textbf {0.71}}$	${\textbf {59.78}}\pm {\textbf {0.76}}$	${\textbf {5.57}}\pm {\textbf {0.08}}$	${\textbf {99.45}}\pm {\textbf {0.01}}$	${\textbf {75.39}}\pm {\textbf {2.86}}$	${\textbf {99.73}}\pm {\textbf {0.04}}$

**Table 3:** Optimization experiments based on SAM-adpter model.All the models were trained based on the 5-fold cross-validation schema, and the slice-level evaluation metrics were reported (mean of the mean in each cv ± std if the mean in each cv) In the Focal Tversky Loss experiments, only $\alpha$ was tunned, while other parameters fixed at $\gamma$=1, $\epsilon$=0.01

Model	Dice(%)	IoU(%)	95%HD	Accuracy	Sensitivity	Specificity
BCE	69.07 ± 0.71	56.29 ± 0.65	5.73 ± 0.07	99.40 ± 0.01	72.76 ± 2.19	99.70 ± 0.04
BCE(75%) + BL(25%)	70.17 ± 0.90	57.44 ± 0.97	5.65 ± 0.03	99.43 ± 0.01	71.98 ± 2.51	99.74 ± 0.03
BCE(50%) + BL(50%)	${\textbf {72.34}}\pm {\textbf {0.71}}$	${\textbf {59.78}}\pm {\textbf {0.76}}$	${\textbf {5.57}}\pm {\textbf {0.08}}$	${\textbf {99.45}}\pm {\textbf {0.01}}$	${\textbf {75.39}}\pm {\textbf {2.86}}$	${\textbf {99.73}}\pm {\textbf {0.04}}$
BCE(25%) + BL(75%)	70.57 ± 1.20	57.85 ± 1.08	5.63 ± 0.10	99.43 ± 0.03	71.81 ± 1.65	99.75 ± 0.04
Dice	70.47 ± 0.72	57.68 ± 0.85	5.68 ± 0.85	99.42 ± 0.01	72.69 ± 1.48	99.73 ± 0.03
Dice(50%) + BL(50%)	70.73 ± 0.76	57.89 ± 0.65	5.66 ± 0.04	99.42 ± 0.01	72.82 ± 1.55	99.74 ± 0.02
Focal Tversky($\alpha$=0.1)	66.82 ± 1.24	52.89 ± 1.18	6.20 ± 0.11	99.15 ± 0.04	85.39 ± 3.02	99.31±0.05
Focal Tversky($\alpha$=0.2)	70.25 ± 0.77	56.93 ± 0.76	5.90 ± 0.09	99.30 ± 0.03	83.80 ± 1.56	99.47±0.06
Focal Tversky($\alpha$=0.4)	71.98 ± 0.43	59.24 ± 0.38	5.64 ± 0.02	99.42 ± 0.01	77.94 ± 1.88	99.67±0.03
Focal Tversky($\alpha$=0.6)	71.15 ± 1.70	58.37 ± 1.17	5.60 ± 0.11	99.45 ± 0.02	72.80 ± 3.02	99.76 ± 0.03
Adapter(M=20)	69.83 ± 0.84	57.33 ± 0.77	5.68 ± 0.06	99.43 ± 0.02	71.73 ± 0.63	99.73 ± 0.03
Adapter(M=50)	70.64 ± 0.50	57.98 ± 0.47	5.60 ± 0.03	99.44 ± 0.01	72.43 ± 1.53	99.74 ± 0.03
Adapter(M=100)	${\textbf {72.34}}\pm {\textbf {0.71}}$	${\textbf {59.78}}\pm {\textbf {0.76}}$	${\textbf {5.57}}\pm {\textbf {0.08}}$	${\textbf {99.45}}\pm {\textbf {0.01}}$	${\textbf {75.39}}\pm {\textbf {2.86}}$	${\textbf {99.73}}\pm {\textbf {0.04}}$
Adapter (M=200)	70.75 ± 0.70	58.12 ± 0.72	5.59 ± 0.04	99.44 ± 0.01	71.80 ± 1.39	99.75 ± 0.02
No CA*, No Pretrain	67.62 ± 1.42	54.32 ± 1.37	5.84 ± 0.06	99.36 ± 0.02	68.23 ± 3.71	99.71 ± 0.05
No CA, Pretrain w/ BHSD	68.49 ± 0.84	55.51 ± 0.73	5.76 ± 0.05	99.38 ± 0.02	69.12 ± 2.23	99.72 ± 0.05
No CA, Pretrain w/ Instance	67.19 ± 0.78	53.82 ± 0.81	5.89 ± 0.08	99.33 ± 0.03	68.57 ± 0.87	99.68 ± 0.03
No CA, Pretrain w/ PyscioNet	68.34 ± 0.74	54.94 ± 0.71	5.80 ± 0.09	99.36 ± 0.02	69.40 ± 0.55	99.71 ± 0.02
No CA, Pretrain w/ All	70.27 ± 1.10	57.27 ± 0.99	5.67 ± 0.08	99.40 ± 0.02	71.88 ± 2.80	99.73 ± 0.04
w/ CA, No pre-train	${\textbf {72.34}}\pm {\textbf {0.71}}$	${\textbf {59.78}}\pm {\textbf {0.76}}$	${\textbf {5.57}}\pm {\textbf {0.08}}$	${\textbf {99.45}}\pm {\textbf {0.01}}$	${\textbf {75.39}}\pm {\textbf {2.86}}$	${\textbf {99.73}}\pm {\textbf {0.04}}$

* CA: Contrast Adjustment

* Focal Tversky: Other parameters were kept constant with $\gamma$=1 and $\epsilon$=0.01

The upper section of Table 3 presents the performance variations across different loss functions, adjustments in loss combination ratios, and hyperparameter tuning for the Focal Tversky Loss. Among the single loss functions, Dice Loss achieved slightly better performance compared to Binary Cross Entropy (BCE). While Focal Tversky Loss produced superior scores when fine-tuned, its reliance on multiple hyperparameters made it prone to overfitting specific settings, compromising generalizability. During the training process, we observed frequent instances of gradient explosion when using Focal Tversky Loss, primarily due to the rapid fluctuations in the Tversky index. This instability often caused the model to fail at the very beginning of training, preventing effective optimization. The underlying issue stems from the nature of the Tversky index, which dynamically adjusts the weighting between false positives and false negatives. Consequently, we only present partial Focal Tversky loss results and excluded Focal Tversky Loss from further combination loss function experiments. Ultimately, the combination of BCE (50%) + BL (50%) yielded the best results, achieving a Dice score of 72.34%, an IoU of 59.78%, and a 95%HD of 5.57. This balanced approach between pixel-wise accuracy and boundary refinement significantly enhanced sensitivity and specificity, making it the optimal choice for our segmentation tasks.

The middle section of Table 3 examines the impact of the adapter block dimension M on the model performance. Our results indicate that setting M=100 achieved the best balance between model complexity and segmentation accuracy. Increasing M beyond this point or decreasing M below this point did not yield further significant improvements, suggesting that M=100 strikes the best balance and provides an optimal trade-off between model complexity and performance.

The lower section of Table 3 compares how the performance with and without pretraining on a public hematoma dataset, as well as the influence of contrast adjustment as a preprocessing step. Without contrast adjustment, pretraining generally improved model performance, except for the INSTANCE dataset, where only a marginal increase in sensitivity was observed. When the model was pretrained on all available public datasets, it showed notable improvements across all evaluation metrics. Interestingly, models trained on contrast-adjusted data consistently outperformed those pretrained on unadjusted datasets. These results indicate that our segmentation task benefits more from focusing on pixels within a specific HU range associated with hematoma, such as blood and brain tissue, rather than relying on features learned from a broader-spectrum dataset. While acute traumatic and spontaneous intracranial hemorrhages appear as hyperdensities with identical HU values on CT scans, they exhibit distinct anatomical distributions and morphologies, which likely influenced model performance.

To validate these findings, we conducted a scan-level evaluation, with results presented in Table S2. Notably, the model that performed best at the slice level was not necessarily the highest-performing at the scan level. For instance, while the BCE + BL combination achieved the highest Dice score in slice-level evaluations, it slightly underperformed compared to Focal Tversky Loss in scan-level evaluations.

To assess the statistical significance of performance variations across different optimization settings, we applied Friedman’s test (Table S4). Although some hyperparameter configurations yielded superior scores, the test did not produce sufficiently low p-values to confirm statistically significant improvements. This indicates that while hyperparameter tuning had an impact, the observed variations may not be consistently meaningful across all settings.

Nevertheless, the overall trend suggests that these optimizations contributed to improved segmentation robustness and consistency across evaluations. By refining loss functions, adjusting adapter block dimensions, and incorporating pretraining strategies, we achieved a more reliable and generalizable segmentation framework.

### Inter-observer variability estimation

**Fig. 4:**
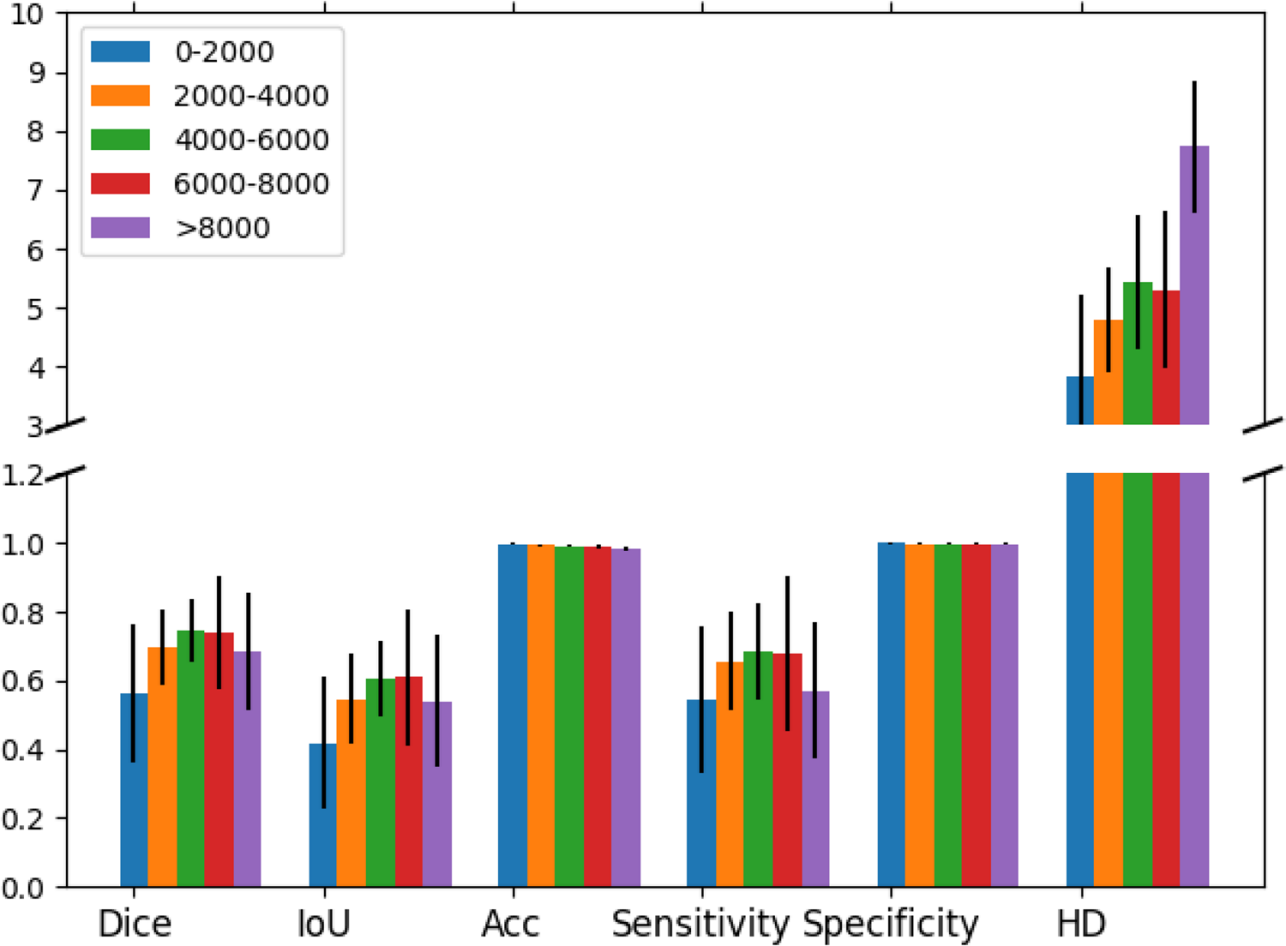
Comparison of peer reviewers’ agreement across different hematoma size groups. The bar graph illustrates the agreement between peer reviewers segmented by hematoma size in CT slices. Hematoma sizes are grouped by pixel count: 0–2000 (blue), 2000–4000 (orange), 4000–6000 (green), 6000–8000 (red), and >8000 (purple). Metrics include Dice coefficient, Intersection over Union (IoU), Accuracy (Acc), Sensitivity, Specificity, and 95% Hausdorff Distance (HD). Error bars represent the standard deviation within each group

To evaluate the consistency and reliability of hematoma segmentation, we assessed inter-observer variability between human reviewers in Fig. 4 and compared it with the performance of our best performing model in Table 4.

The comparison between human annotations and the model predictions resulted in a Dice of 67.20% and an IoU of 54.34%. The 95%HD was 5.75, indicating reasonable boundary agreement and the 99.61% sensitivity demonstrates that the model performed well in detecting hematomas, with a higher rate of true positives and a relatively low rate of false positives.

The variability between human reviewers showed slightly lower Dice and IoU comparing the human-model difference. The 95%HD was lower at 4.70, suggesting better boundary agreement between human reviewers than between human and model. However, the overall accuracy was lower at 95.90%, with sensitivity and specificity at 59.70% and 96.31%, respectively. These results indicate that while human reviewers generally agree with the boundary of mutual hematoma findings, there are notable differences/missing in hematoma appearance. Additionally, when we break down the hematoma size into different groups as shown in Fig. 4, we observe that both reviewers had better agreement on medium-sized hematomas. The Dice, IoU, and sensitivity dropped when the hematoma size was either too large or too small. Larger hematoma sizes were associated with relatively larger absolute volume differences, indicating that discrepancies in segmentation become more pronounced as the size of the hematoma increases.

**Fig. 5:**
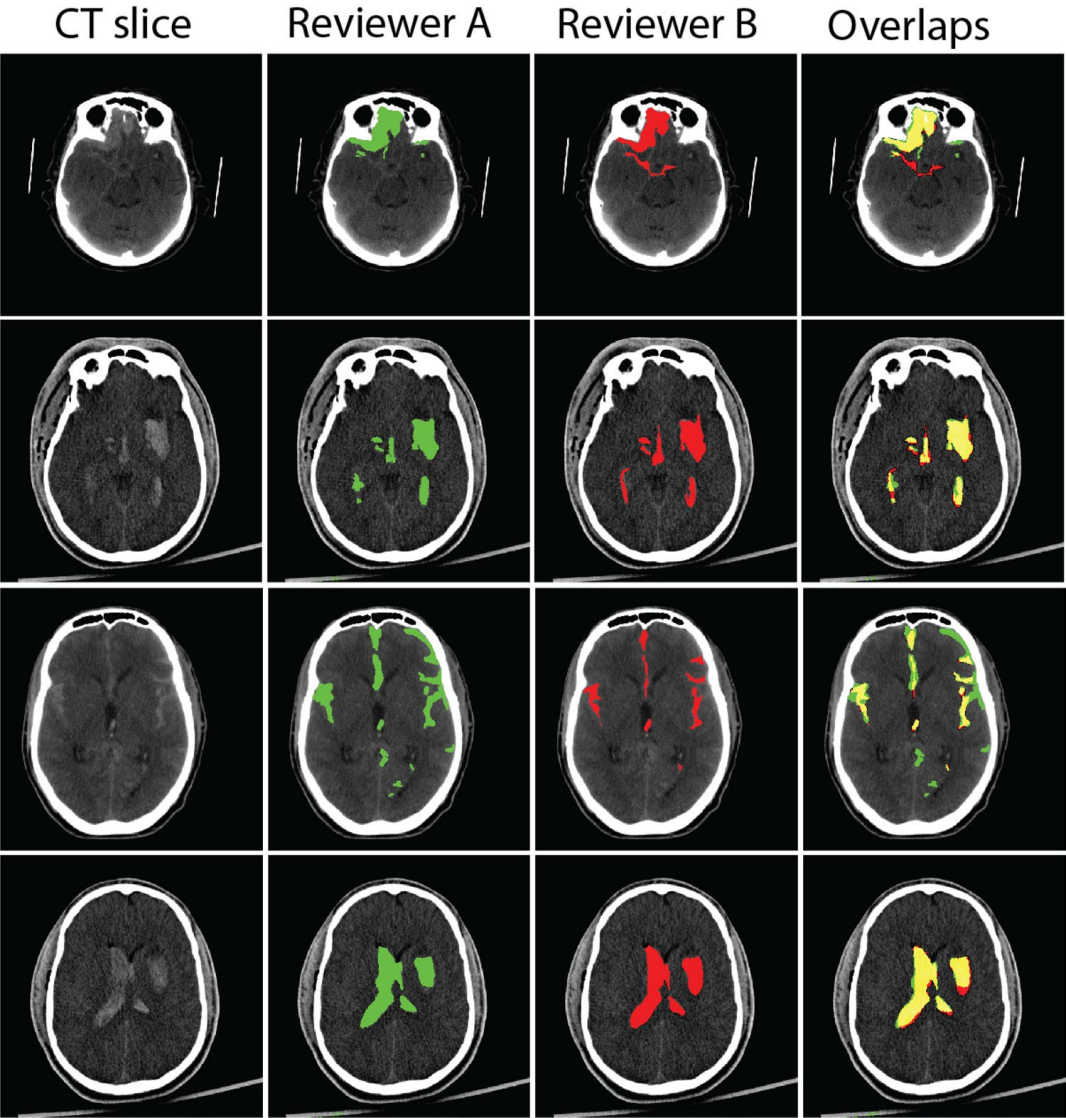
Comparison of human error in hematoma segmentation between two reviewers. Each row represents a selected slice from the peer-reviewed dataset. The first column shows the contrast-adjusted CT slices with a window level of [0–140] HU. The middle columns display the manual segmentations by Reviewer A (in green) and Reviewer B (in red), respectively. The last column presents the overlap of annotations from both reviewers, with the intersecting regions shown in yellow and the non-overlapping annotations retaining their original colors (green for Reviewer A and red for Reviewer B)

These findings underscore the importance of considering hematoma size when evaluating inter-observer variability, as agreement levels may vary significantly with size, impacting the reliability of manual segmentation in clinical settings (see Fig. 5).

**Table 4:** Comparison between model vs human error and human error on peer reviewed dataset. Results presented as mean of slice-level evaluation metrics

Model	Dice(%)	IoU(%)	95%HD	Accuracy	Sensitivity	Specificity
Human vs Model	67.20	54.34	5.75	99.35	74.89	99.61
Human vs Human	63.79	49.87	4.70	95.90	59.70	96.31

### Evaluation on external validation dataset

To assess the generalizability of our model, we conducted external validation using the HemSeg-200 dataset, which consists of 222 scans with voxel-level hemorrhage annotations. This dataset encompasses a diverse range of cases, capturing variations in hemorrhage patterns across different patients. As summarized in Table 5, the SAM-Adapter model achieved the highest segmentation performance, with a Dice score of 81.10%, an IoU of 70.35%, and a 95%HD of 38.91. Compared to UNet and nnUNet, which serve as benchmark models, SAM-Adapter demonstrated superior segmentation accuracy, achieving the highest Dice and IoU scores while maintaining consistent performance across external validation data.

Notably, nnUNet exhibited strong overall performance, particularly excelling in 95%HD, where it outperformed both UNet and SAM-Adapter. This advantage is likely due to nnUNet’s 3D architecture, which effectively captures spatial relationships across volumetric data, resulting in enhanced boundary precision and a reduced Hausdorff Distance in three dimensions. However, while nnUNet demonstrated superior spatial consistency, the SAM-Adapter model achieved the best overall segmentation accuracy, as reflected in its higher Dice and IoU scores.

**Table 5:** Validation of segmentation performance across different models on the HemSeg-200 dataset

Model	Dice(%)	IoU(%)	95%HD
UNet[41]	63.70 (55.82,71.10)	51.40 (44.26,58.48)	83.48 (51.66,88.37)
nnUNet[41]	78.99 (73.21,83.86)	68.49 (62.05,74.25)	22.16 (9.19,38.20)
SAM-Adapter	81.10(76.47,85.07)	70.35 (64.89,75.30)	38.91 (25.64,54.40)

Results are reported as the scan-level mean with a 95% bootstrap confidence interval, computed on the independent test set

## Discussion

The findings of this study highlight the potential clinical impacts of utilizing advanced segmentation models for hematoma estimation in TBI patients. The automatic and precise segmentation of hematoma lesions with the optimized SAM-Adapter model significantly enhances the accuracy and efficiency of diagnosing TBI. This capability is crucial for timely and effective treatment planning, as precise identification of hematoma boundaries and volumes can guide surgical interventions and other therapeutic strategies. The high sensitivity and specificity also suggest the ability to identify hematomas with reduced likelihood of missed diagnoses, and eventually improving patient outcomes.

From a computational perspective, SAM models generally require greater computational resources than conventional neural networks. Our implementation, however, addresses this by leveraging parameter-efficient transfer learning (PETL), which significantly reduces the number of trainable parameters while maintaining high segmentation performance. Specifically, we freeze the original SAM parameters and fine-tune only the adapter layers, which contain fewer than 4 million parameters compared to the 86 million in the base model. This approach ensures computational feasibility while preserving model effectiveness, making it more adaptable to resource-limited clinical environments.

Despite the promising results, some limitations and challenges need to be addressed. A key limitation in our current approach is its reliance on 2D segmentation methods, which struggle to effectively utilize the spatial information available in 3D CT imaging. While our results show that SAM-Adapter outperforms nnUNet, a leading 3D benchmark model, further improvements can be achieved by integrating 3D spatial context. Another challenge is the accurate segmentation of small lesions and those with irregular shapes, which might be more clinically significant despite contributing minimally to overall evaluation scores. Our study suggests that human reviewers and models are likely to struggle with small lesion segmentation, underscoring the need for targeted enhancements.

A critical aspect of hematoma assessment is the ability to distinguish between different subtypes, as each may have distinct clinical implications. While clinicians can visually differentiate these subtypes in CT scans, developing automatic methods to distinguish them will be a valuable future direction. Our study does not directly classify hematoma subtypes, but the improved segmentation accuracy provides a strong foundation for future extensions incorporating multi-class differentiation.

To further enhance the performance and clinical applicability of hematoma segmentation models, several future directions are proposed. One crucial area for future development is the incorporation of hybrid 2D-3D architectures or volumetric processing techniques to better capture complex spatial relationships in CT scans. While our model outperforms existing 3D architectures in segmentation accuracy, leveraging 3D spatial information more effectively could further enhance volumetric consistency and boundary delineation. Additionally, implementing auxiliary network branches or attention mechanisms specifically designed to highlight underrepresented lesions could help mitigate the small hematoma detection issue by refining the segmentation of difficult cases. To ensure robust generalization, future work should involve training on larger and more diverse datasets while integrating annotations from multiple experts. This approach would reduce biases introduced by individual annotators and enhance the model’s adaptability to different clinical settings. Furthermore, quantifying hematoma volume using our proposed segmentation method and correlating these measurements with neurological outcomes would provide valuable clinical validation, reinforcing the practical utility of automated segmentation in guiding treatment decisions.

By addressing these challenges, future research can build upon the strengths of our approach, advancing automated hematoma segmentation toward greater accuracy, reliability, and clinical impact.

## Supplementary Information

Below is the link to the electronic supplementary material.

Supplementary file 1.

## Data Availability

The medical imaging datasets analyzed during the current study are not publicly available due to restrictions related to patient privacy and confidentiality. The data contains sensitive and protected health information governed by applicable privacy laws and institutional policies.

## References

[1] Maas AI, et al. Traumatic brain injury: progress and challenges in prevention, clinical care, and research. Lancet Neurol. 2022;21:1004–60. 36183712 10.1016/S1474-4422(22)00309-X PMC10427240

[2] Blennow K, et al. Traumatic brain injuries. Nat Rev Dis Primers. 2016;2:1–19. 10.1038/nrdp.2016.84 27853132

[3] Khellaf A, Khan DZ, Helmy A. Recent advances in traumatic brain injury. J Neurol. 2019;266:2878–89. 31563989 10.1007/s00415-019-09541-4 PMC6803592

[4] Wilson L, et al. The chronic and evolving neurological consequences of traumatic brain injury. Lancet Neurol. 2017;16:813–25. 28920887 10.1016/S1474-4422(17)30279-X PMC9336016

[5] Rajaei F, Cheng S, Williamson CA, Wittrup E, Najarian K. Ai-based decision support system for traumatic brain injury: a survey. Diagnostics. 2023;13:1640. 37175031 10.3390/diagnostics13091640 PMC10177859

[6] Currie S, et al. Imaging assessment of traumatic brain injury. Postgrad Med J. 2016;92:41–50. 26621823 10.1136/postgradmedj-2014-133211

[7] Zimmerman RD, Maldjian JA, Brun N, Horvath B, Skolnick B. Radiologic estimation of hematoma volume in intracerebral hemorrhage trial by CT scan. Am J Neuroradiol. 2006;27:666–70. 16552014 PMC7976993

[8] Mutch CA, Talbott JF, Gean A. Imaging evaluation of acute traumatic brain injury. Neurosurg Clin. 2016;27:409–39. 10.1016/j.nec.2016.05.011 PMC5027071 27637393

[9] Foreman B, Lissak IA, Kamireddi N, Moberg D, Rosenthal ES. Challenges and opportunities in multimodal monitoring and data analytics in traumatic brain injury. Curr Neurol Neurosci Rep. 2021;21:1–9. 10.1007/s11910-021-01098-y PMC7850903 33527217

[10] Abdollahifard S, Farrokhi A, Mowla A. Application of deep learning models for detection of subdural hematoma: a systematic review and meta-analysis. J NeuroIntervent Surg. 2023;15:995–1000. 10.1136/jnis-2022-019627 36418163

[11] Cho J, et al. Improving sensitivity on identification and delineation of intracranial hemorrhage lesion using cascaded deep learning models. J Dig Imag. 2019;32:450–61. 10.1007/s10278-018-00172-1 PMC6499861 30680471

[12] Yao H, Williamson C, Gryak J, Najarian K. Automated hematoma segmentation and outcome prediction for patients with traumatic brain injury. Artif Intell Med. 2020;107: 101910. 32828449 10.1016/j.artmed.2020.101910

[13] Chang PD, et al. Hybrid 3d/2d convolutional neural network for hemorrhage evaluation on head CT. Am J Neuroradiol. 2018;39:1609–16. 30049723 10.3174/ajnr.A5742 PMC6128745

[14] Alis D, et al. A joint convolutional-recurrent neural network with an attention mechanism for detecting intracranial hemorrhage on noncontrast head CT. Sci Rep. 2022;12:2084. 35136123 10.1038/s41598-022-05872-x PMC8826390

[15] Farzaneh N, et al. Automated segmentation and severity analysis of subdural hematoma for patients with traumatic brain injuries. Diagnostics. 2020;10:773. 33007929 10.3390/diagnostics10100773 PMC7600198

[16] Ye H, et al. Precise diagnosis of intracranial hemorrhage and subtypes using a three-dimensional joint convolutional and recurrent neural network. Eur Radiol. 2019;29:6191–201. 31041565 10.1007/s00330-019-06163-2 PMC6795911

[17] Kellogg RT, et al. Segmentation of chronic subdural hematomas using 3d convolutional neural networks. World Neurosurg. 2021;148:e58–65. 33359736 10.1016/j.wneu.2020.12.014

[18] Inkeaw P, et al. Automatic hemorrhage segmentation on head CT scan for traumatic brain injury using 3d deep learning model. Comput Biol Med. 2022;146: 105530. 35460962 10.1016/j.compbiomed.2022.105530

[19] Kirillov A, et al. Segment anything. arXiv preprint. 2023. arXiv:2304.02643.

[20] Huang Y, et al. Segment anything model for medical images? Med Image Analy. 2024;92: 103061. 10.1016/j.media.2023.103061 38086235

[21] Ma J, Wang B. Segment anything in medical images. arXiv preprint. 2023. arXiv:2304.12306.

[22] Houlsby N, et al. Parameter-efficient transfer learning for NLP. 2019.

[23] Wu J, et al. Medical sam adapter: Adapting segment anything model for medical image segmentation. arXiv preprint. 2023. arXiv:2304.12620. 10.1016/j.media.2025.103547 40121809

[24] DenOtter TD, Schubert J. Hounsfield unit. Teasure Island: StatPearls; 2023. 31613501

[25] Alexey D. An image is worth 16x16 words: transformers for image recognition at scale. arXiv preprint. 2020. arXiv:2010.11929.

[26] He K. et al. Masked autoencoders are scalable vision learners. 2022.

[27] Tajbakhsh N, et al. Convolutional neural networks for medical image analysis: full training or fine tuning? IEEE Trans Med Imag. 2016;35:1299–312. 10.1109/TMI.2016.2535302 26978662

[28] Ghafoorian M. et al. Transfer learning for domain adaptation in MRI: application in brain lesion segmentation. 2017.

[29] Lee J, Tang R, Lin J. What would elsa do? freezing layers during transformer fine-tuning. arXiv preprint. 2019. arXiv:1911.03090.

[30] Shen Z, Liu Z, Qin J, Savvides M, Cheng K-T. Partial is better than all: revisiting fine-tuning strategy for few-shot learning. 2021.

[31] Han Z, Gao C, Liu J, Zhang SQ. et al. Parameter-efficient fine-tuning for large models: a comprehensive survey. arXiv preprint. 2024. arXiv:2403.14608.

[32] Dutt R, Ericsson L, Sanchez P, Tsaftaris SA, Hospedales T. Parameter-efficient fine-tuning for medical image analysis: the missed opportunity. arXiv preprint. 2023. arXiv:2305.08252.

[33] Chen T, et al. Sam-adapter: Adapting segment anything in underperformed scenes. 2023.

[34] Wang Y, et al. Samihs: adaptation of segment anything model for intracranial hemorrhage segmentation. 2024.

[35] Ronneberger O, Fischer P, Brox T. U-net: convolutional networks for biomedical image segmentation. 2015.

[36] Isensee F, Jaeger PF, Kohl SA, Petersen J, Maier-Hein KH. nnu-net: a self-configuring method for deep learning-based biomedical image segmentation. Nat Methods. 2021;18:203–11. 33288961 10.1038/s41592-020-01008-z

[37] Wright DW, et al. Very early administration of progesterone for acute traumatic brain injury. N Engl J Med. 2014;371:2457–66. 25493974 10.1056/NEJMoa1404304 PMC4303469

[38] Wu B, et al. Bhsd: A 3d multi-class brain hemorrhage segmentation dataset. 2023.

[39] Li X, et al. The state-of-the-art 3d anisotropic intracranial hemorrhage segmentation on non-contrast head ct: The instance challenge. arXiv preprint. 2023. arXiv:2301.03281.

[40] Hssayeni M, et al. Computed tomography images for intracranial hemorrhage detection and segmentation. Intracr Hemorrhage Segment Using Deep Convol Model Data. 2020;5:14.

[41] Song C, et al. Hemseg-200: a voxel-annotated dataset for intracerebral hemorrhages segmentation in brain ct scans. arXiv preprint. 2024. arXiv:2405.14559.

[42] Li X, et al. Hematoma expansion context guided intracranial hemorrhage segmentation and uncertainty estimation. IEEE J Biomed Health Inform. 2021;26:1140–51. 10.1109/JBHI.2021.3103850 34375295

[43] Flanders AE, et al. Construction of a machine learning dataset through collaboration: the RSNA 2019 brain CT hemorrhage challenge. Radiol Artif Intell. 2020;2: e190211. 33937827 10.1148/ryai.2020190211 PMC8082297

[44] Müller D, Soto-Rey I, Kramer F. Towards a guideline for evaluation metrics in medical image segmentation. BMC Res Notes. 2022;15:210. 35725483 10.1186/s13104-022-06096-y PMC9208116

[45] Taha AA, Hanbury A. Metrics for evaluating 3d medical image segmentation: analysis, selection, and tool. BMC Med Imag. 2015;15:1–28. 10.1186/s12880-015-0068-x PMC4533825 26263899

[46] Rainio O, Teuho J, Klén R. Evaluation metrics and statistical tests for machine learning. Sci Rep. 2024;14:6086. 38480847 10.1038/s41598-024-56706-x PMC10937649

