# Supplementary material for: Adapting segment anything model for hematoma segmentation in traumatic brain injury
Source: Discov Imaging. 2025 May 26;2(1):6. doi: 10.1007/s44352-025-00011-4 (PMC12106135; doi:10.1007/s44352-025-00011-4)
Supplement: Supplementary file 1 [file 44352_2025_11_MOESM1_ESM.pdf]

Table S 1 Segmentation performance comparison across different models (Scan Level). All models were trained using a 5-fold cross-validation schema, and scan-level evaluation metrics are reported on the test set as the mean of the fold-wise means ± the mean of the fold-wise standard deviation.

Model	Dice(%)	IoU(%)	95%HD	VS(%)
UNet	41.64±31.63	31.39±25.43	124.41±50.90	61.30±38.23
MultiView	40.95±30.54	30.41±24.41	121.76±53.63	57.69±38.81
nnUNet	68.82±15.94	54.54±16.92	110.27±67.62	88.28±11.42
SAM	43.63±17.12	29.57±13.80	136.69±49.09	74.19±20.59
**SAM-Adapter**	**75.51±11.45**	**61.92±13.65**	**94.96±47.76**	**92.05±7.49**

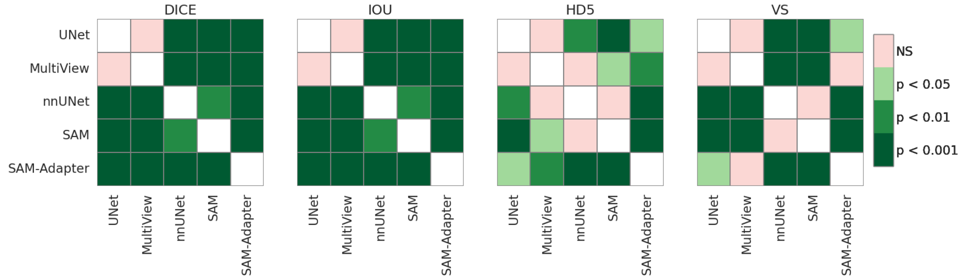

Figure S 1 Nemenyi post-hoc test assessing the statistical significance of model performance differences.

.
Table S 2 Optimization Experiments Based on SAM-Adpter Model (Scan Level). All the models were trained based on the 5-fold cross-validation schema, and the scan-level evaluation metrics are reported on the test set as the mean of the fold-wise means ± the mean of the fold-wise standard deviation.)

Model	Dice(%)	IoU(%)	95%HD	VS(%)
BCE	73.89±10.35	59.59±12.16	96.01±49.43	91.25±8.09
BCE(75%) + BL(25%)	73.81±11.28	59.69±13.22	90.84±47.91	91.33±7.78
BCE(50%) + BL(50%)	75.51±11.45	61.92±13.65	94.96±47.76	92.05±7.49
BCE(25%) + BL(75%)	74.22±11.54	60.28±13.63	94.53±46.99	92.31±6.83
Dice	74.47±11.17	60.50±13.14	85.23±47.95	92.15±7.70
Dice(50%) + BL(50%)	74.24±11.57	60.30±13.65	88.05±49.65	92.75±6.60
Focal Tversky(α=0.1)	69.68±11.54	54.64±12.66	105.57±44.01	80.62±12.68
Focal Tversky(α=0.2)	74.13±10.56	59.94±12.37	97.89±51.94	86.24±9.44
Focal Tversky(α=0.4)	75.65±10.75	61.96±13.03	92.15±45.33	91.71±6.43
Focal Tversky(α=0.6)	74.46±11.96	60.64±13.80	92.54±47.93	91.51±8.79
Adapter(M=20)	72.86±13.16	58.86±14.79	93.65±46.02	91.01±9.73
Adapter(M=50)	74.68±11.37	60.82±13.49	92.18±52.56	92.67±6.27
Adapter(M=100)	75.51±11.45	61.92±13.65	94.96±47.76	92.05±7.49
Adapter (M=200)	74.71±11.59	60.90±13.66	89.29±47.28	92.38±6.64
No CA*, No Pretrain	71.32±12.52	56.84±14.23	106.99±50.89	91.11±6.23
No CA, Pretrain w/ BHSD	72.15±12.59	57.83±14.11	101.31±48.34	91.27±6.81
No CA, Pretrain w/ Instance	69.96±13.13	55.30±14.51	109.14±49.43	90.29±6.52
No CA, Pretrain w/ PhysioNet	71.18±12.05	56.53±13.58	110.97±52.89	91.91±5.47
No CA, Pretrain w/ All	73.35±11.74	59.19±13.45	97.53±52.78	91.65±6.12
w/ CA, No pre-train	75.51±11.45	61.92±13.65	94.96±47.76	92.05±7.49

* CA: Contrast Adjustment

* Focal Tversky: Other parameters were kept constant with γ=1 and ϵ=0.01

.

Table S 3 Friedman's Test on different model architectures(Scan Level)

Metrics	Dice		IoU		95%HD		VS(%)	
Model	Statistic	p-value	Statistic	p-value	Statistic	p-value	Statistic	p-value
UNet<br>MultiView<br>nnUNet<br>SAM<br>SAM-Adapter	47.21	3.13e-10***	47.21	3.13e-10***	14.92	1.89e-3***	31.16	7.87e-7***

* p-value <0.05
** p-value <0.01
*** p-value <0.001

.

Table S 4 Friedman's Test on Model Optimization Experiments (Scan Level)

Metrics	Dice		IoU		95%HD		VS(%)	
Model	Statistic	p-value	Statistic	p-value	Statistic	p-value	Statistic	p-value
BCE<br>BCE(75%) + BL(25%)<br>BCE(50%) + BL(50%)<br>BCE(25%) + BL(75%)<br>Dice<br>Dice(50%) + BL(50%)<br>Focal Tversky(α=0.1)<br>Focal Tversky(α=0.2)<br>Focal Tversky(α=0.4)<br>Focal Tversky(α=0.6)	5.47	0.14	5.47	0.14	1.26	0.73	0.57	0.90
Adapter(M=20)<br>Adapter(M=50)<br>Adapter(M=100)<br>Adapter (M=200)	4.73	0.14	4.73	0.19	0.14	0.99	0.32	0.96
No CA*, No Pretrain<br>No CA, Pretrain w/ BHSD<br>No CA, Pretrain w/ Instance<br>No CA, Pretrain w/ BCIHM<br>No CA, Pretrain w/ All<br>w/ CA, No pre-train	5.40	0.14	5.40	0.14	1.29	0.73	1.44	0.70

* CA: Contrast Adjustment
* Focal Tversky: Other parameters were kept constant with γ=1 and ϵ=0.01
